# The Pivotal Role of One-Carbon Metabolism in Neoplastic Progression During the Aging Process

**DOI:** 10.3390/biom14111387

**Published:** 2024-10-31

**Authors:** Avisek Majumder, Shabana Bano, Kasturi Bala Nayak

**Affiliations:** 1 Department of Medicine, University of California, San Francisco, CA 94158, USA; 2 Quantitative Biosciences Institute, Department of Medicine, University of California, San Francisco, CA 94158, USA

**Keywords:** cancer prevention, anti-cancer therapeutics, homocysteine, methionine addition, serine biosynthesis, glycine biosynthesis, folate, senescence, antiproliferative response, redox statuses, oxidative stress, hyperhomocysteinemia, glutathione, hydrogen sulfide (H_2_S), antioxidants, diet, lifestyle, cancer therapy, epigenetics, inflammation, Warburg effect, chemotherapeutic agents

## Abstract

One-carbon (1C) metabolism is a complex network of metabolic reactions closely related to producing 1C units (as methyl groups) and utilizing them for different anabolic processes, including nucleotide synthesis, methylation, protein synthesis, and reductive metabolism. These pathways support the high proliferative rate of cancer cells. While drugs that target 1C metabolism (like methotrexate) have been used for cancer treatment, they often have significant side effects. Therefore, developing new drugs with minimal side effects is necessary for effective cancer treatment. Methionine, glycine, and serine are the main three precursors of 1C metabolism. One-carbon metabolism is vital not only for proliferative cells but also for non-proliferative cells in regulating energy homeostasis and the aging process. Understanding the potential role of 1C metabolism in aging is crucial for advancing our knowledge of neoplastic progression. This review provides a comprehensive understanding of the molecular complexities of 1C metabolism in the context of cancer and aging, paving the way for researchers to explore new avenues for developing advanced therapeutic interventions for cancer.

## 1. Introduction

Cancer cells are found to maintain a different metabolism status than normal cells to support their uncontrolled proliferation and survival [1,2,3]. This altered metabolism of cancer cells was first observed by Dr. Otto Heinrich Warburg, who was awarded the Nobel Prize in 1931 for his discovery [4]. Dr. Warburg noticed that, unlike normal cells, most cancer cells produce energy not predominantly through the citric acid cycle and oxidative phosphorylation (OXPHOS) in the mitochondria but through glycolysis followed by lactic acid fermentation in the cytosol (this phenomenon is known as the ‘Warburg effect’) [5]. Although the precise metabolic advantage of the Warburg effect is still unclear, many hypotheses have been proposed, which include rapid ATP production, one-carbon metabolism, a defect in the mitochondria/OXPHOS, and reduced reactive oxygen species (ROS) production in cancer cells [4,6,7,8]. With or without the Warburg effect, cancer cells require one carbon (1C) unit for their methylation process; the biosynthesis of nucleotides, lipids, and proteins; and to maintain their redox status [9]. This increased glucose utilization has been exploited in the positron emission tomography (PET) imaging of tumor growth and metastases [10]; however, targeting any specific signaling intermediates of this pathway remains elusive for cancer treatment.

**Figure 1:**
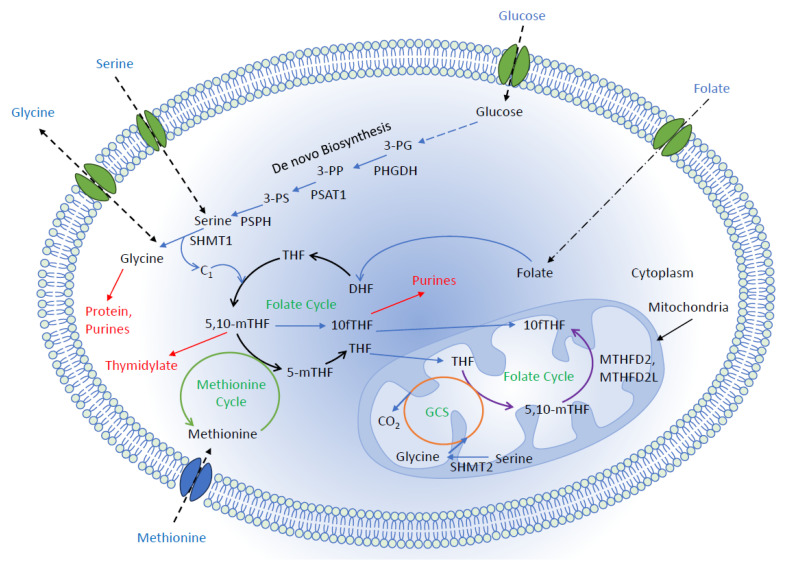
This cartoon diagram illustrates the enzymatic reactions and compartmentalization of 1C metabolism. Cells utilize 1C units from methionine, serine, and glycine to produce various compounds that work as building blocks for the biosynthesis of nucleic acids and proteins, regulate methylation reactions, and help maintain a cellular redox status. Serine and glycine can enter cells from the outside or be synthesized de novo from the glycolysis intermediate, 3-phosphoglycerate (3-PG). Methionine and folate always come from diet, are carried over the methionine cycle, and can operate in both the cytoplasm and mitochondria (all abbreviations are given at the end).

The term “one-carbon metabolism” is used to refer to a complex network of metabolic reactions that are closely related to producing 1C units (as methyl groups) and subsequently utilizing them for the biosynthesis of important anabolic precursors and methylation reactions [11]. Tetrahydrofolate (THF) is a universal 1C acceptor, whereas serine and glycine work as 1C unit donors [12]. In this process, THF converts to methyl-THF, which is subsequently used for purine synthesis and the re-methylation of homocysteine (via the methionine-recycling pathway) [13]. One-carbon signaling pathways are regulated through the de novo serine synthesis pathway and the mitochondrial 1C pathways, as shown in Figure 1 [12,14]. All cells require 1C units for nucleotide synthesis, methylation pathways, NADH/NADPH production, and antioxidant production (such as glutathione and hydrogen sulfide) [11]. Specifically, for cancer cells, these processes are a fundamental requirement for consistent energy production, cell proliferation, and growth [15,16].

From a historical perspective, enhanced 1C metabolism in cancer cells was identified in 1948 by Sydney Farber [17]. Sydney Farber noticed that a reduction in dietary folate decreases the number of leukemic cells in children with acute lymphoblastic leukemia, so he tried using the folic acid antagonist aminopterin to treat these cancer patients and was able to achieve temporary remission [18]. This effort from Sydney Farber (who is regarded as the father of modern chemotherapy) led to the development of other chemotherapeutic agents against different types of cancer [19]. Although many drugs were later developed that target the folate and methionine cycles, they showed many deadly side effects due to the importance of these pathways in normal healthy tissues [20]. Nevertheless, many recent studies have shown further insights into 1C metabolism in the context of cancer vs. aging [21,22], suggesting future possibilities for developing newer therapeutic strategies for cancer.

Although studies have shown that biological age is a well-known risk factor for malignancy, the precise mechanism of this interrelated process is still poorly understood [23]. In this regard, epidemiological data suggest that lifestyle factors (such as leanness, a plant-based diet, voluntary physical activity, and the avoidance of environmental mutagens) can slow the aging process and also reduce the probability of developing malignant diseases [24,25]. Among the hypotheses that have been proposed to explain why neoplastic transformation increases with aging, impaired 1C metabolism is one of them (elaborated in detail in Section 6). One-carbon metabolism supports the growth and proliferation of cancer cells and is pivotal to the aging process, mainly via oxidative DNA damage, lipid peroxidation, and epigenetic alteration [26,27]. This review revitalizes the idea of targeting 1C metabolism for cancer treatment in the context of the intricate molecular mechanisms of different signaling pathways associated with aging. We elaborate on the differential utilization of 1C metabolites between the cancer vs. aging processes and how these processes are regulated inside a cell. It is the strength of basic science that determines whether translational therapeutic efforts will have a significant impact. This review work will likely help many researchers in academia and industry to develop next-generation anti-cancer therapeutics that disrupt 1C metabolism pathways in a better way with minimal side effects.

## 2. Utilization of the 1C Unit in Various Metabolic Pathways

### 2.1. Methionine Metabolism Pathways

**Figure 2:**
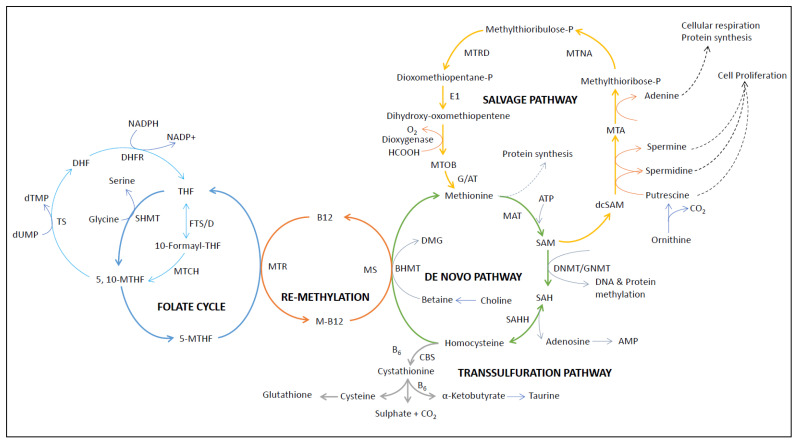
One-carbon (1C) metabolism through the methionine cycle and folate cycle and its utilization in other closely linked pathways (like polyamine synthesis and the transsulfuration pathway) (all abbreviations are given at the end).

Methionine is one of the essential amino acids that are not synthesized in the mammalian system [28]. Therefore, an indispensable source of methionine is from our diet [9,29]. Methionine enters cells via different methionine transporters (like SLC1A5, SLC7A5, SLC7A8, SLC7A6, SLC7A7, SLC38A1, and SLC38A2) [30]. This amino acid (methionine) is not only used for protein formation but is also metabolized through the methionine cycle, as shown in Figure 2. In the first step of the methionine cycle, methionine adenosyltransferase (MAT) converts methionine to S-adenosylmethionine (SAM) with the use of one ATP [30,31]. Interestingly, SAM is the only methyl donor that gives a methyl group to nearly all methylation reactions in the body [32]. After transferring the methyl group via various transmethylation reactions with the help of different methyltransferases (such as DNA methyltransferases, histone methyltransferases, etc.), SAM irreversibly converts to S-adenosylhomocysteine (SAH or AdoHcy) [33]. SAH is the metabolic precursor of homocysteine (Hcy) and also acts as a negative regulator of all methyltransferases mentioned above [34]. Then, SAH is reversibly hydrolyzed to Hcy and adenosine (Ado) by SAH hydrolase (AHCY or SAHH) [35]. It is important to notice that the hydrolysis of SAH to Hcy and adenosine is the only reversible reaction in the cycle. Thermodynamically, it favors the condensation of Hcy and adenosine to SAH. However, this cycle can only proceed if Hcy is efficiently removed or transformed [36]. High total Hcy levels (>15 μmol/L) in plasma, also known as hyperhomocysteinemia (HHcy), have been associated with various types of diseases, including cancer [11,31,37]. After the production of Hcy, it bifurcates into the transsulfuration and remethylation pathways [38]. In normal physiological conditions, 50% of Hcy goes to the transsulfuration pathway, and another 50% of Hcy is re-methylated back to methionine with the help of methionine synthase (5-methyltetrahydrofolate-homocysteine methyltransferase; MTR or MS) or betaine-homocysteine methyltransferase (BHMT) to complete the methionine cycle (Figure 2) [15]. There are different forms of Hcy found in blood circulation, which consist of around 1% as free thiol, 70–80% present and bound to plasma proteins, and the remaining 20–30% present as forms homo/heterodimerized with other thiols [15]. Due to its exceptionally low pKa of 6.7, Hcy-thiolactone remains mostly neutral in the blood and is primarily cleared by the kidneys (~1% is reabsorbed, and over 95% is excreted by the kidneys) [39]. In the transsulfuration pathway, Hcy converts to cystathionine with the help of cystathionine-β-synthase (CBS); cystathionine is then converted to cysteine by cystathionine γ-lyase (CSE) for the production of glutathione (GSH) [40]. In both of these conversions (Hcy to cystathionine and cystathionine to cysteine), H_2_S is produced as a byproduct, which has recently been shown to have an antioxidative, anti-inflammatory, and anti-apoptotic role in our body [41]. In addition to methylation reactions, SAM can also be used for polyamine synthesis, as shown in Figure 2. During polyamine synthesis, S-methyl-5′-thioadenosine (MTA) is produced as a byproduct, which can be further recycled back to methionine via the salvage pathway [42,43].

### 2.2. Serine and Glycine Metabolism Pathways

Serine and glycine are both non-essential amino acids that either come from the diet or can be synthesized inside our body (via de novo biosynthesis) from glycolytic intermediate, 3-PG [44]. In the glycolysis pathway, 3-PG is first oxidized to 3-phosphate hydroxy-pyruvate by phosphoglycerate dehydrogenase (PHGDH), then catalyzed to 3-phosphoserine by phosphoserine aminotransferase (PSAT1), and, finally, dephosphorylated to serine by 1-3-phosphoserine phosphatase (PSPH) [45]. Serine and glycine can be mutually converted via hydroxymethyltransferases (cytosolic: SHMT1 and mitochondrial: SHMT2) [46]. During serine-to-glycine conversion, serine gives 1C unit to THF to produce 5,10-methyleneTHF [47]. Alternatively, glycine can also be converted to serine at the cost of 1C unit from 5,10-methyleneTHF [48,49].

This 1C unit generated from serine and glycine can be used as a precursor for purine and pyrimidine nucleotide synthesis [50]. The first precursor for purine biosynthesis is ribose-5-phosphate, which is synthesized from the pentose phosphate pathway (PPP) [51]. Then, in a series of steps, two 1C units and one glycine unit are added to form inosine monophosphate (IMP), which is then further converted to different purine nucleotides (adenosine and guanosine monophosphate) [52]. However, during pyrimidine biosynthesis, a 1C unit is used in methylation reactions using methylene-tetrahydrofolate (methylene-THF) as the methyl donor, where deoxyuridine monophosphate (dUMP) is converted to deoxythymidine monophosphate (dTMP) with the help of thymidylate synthase (TYMS) [53]. In this reaction, methylene-THF is converted to dihydrofolate (DHF) and then reduced back to THF with the help of dihydrofolate reductase (DHFR) via the folate cycle (Figure 2) [54]. In addition to purine and pyrimidine nucleotide synthesis, glycine also acts as a source of 1C units in the glycine cleavage system (GCS), as shown in Figure 1. GCS is localized in mitochondria, where glycine gives its 1C unit to THF to produce methylene-THF and, as a consequence, also produces NADH from NAD^+^.

## 3. Regulation of 1C Metabolism Under Different Nutrient Statuses

**Figure 3:**
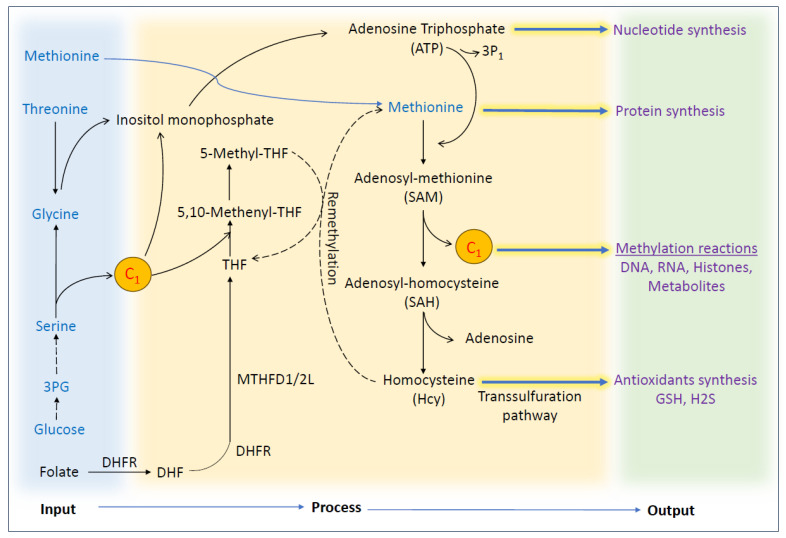
Schematic representation showing the inputs of 1C units from dietary sources and their processing and utilization in different biosynthesis processes as output. In this process, methionine, glucose, serine, and glycine can be used as inputs to carry over 1C metabolism. Serine can be obtained from the diet or produced from glucose via the de novo process. Folate from the diet is converted to THF, which accepts a 1C unit during the folate cycle. Then, serine is broken down into glycine, producing a 1C unit which combines with THF to form methylene-THF. Methionine from the diet can be used to produce SAM, which is subsequently used for methylation reactions and cellular antioxidant production. Different outputs from 1C metabolism also act as building blocks for the cellular biosynthesis of DNA, RNA, and protein.

Methionine, glycine, and serine are the three main precursors of 1C metabolism. Additionally, dietary micronutrients such as folate and vitamins B12 and B6 contribute to 1C metabolism, as shown in Figure 2. These dietary sources act as inputs, which are further processed into different compounds that are required for protein synthesis, nucleotide synthesis, methylation reaction, and antioxidant production (Figure 3) [55]. Although historically folate and other B vitamins were thought to act as chemoprotectants, later epidemiological studies suggest that their effect may increase the risk of cancer [56,57]. Research indicates that higher serum levels of folate may potentially increase the risk of certain cancers, including lung cancer [58], breast cancer [59], and colorectal cancer [60]. Similarly, elevated vitamin B12 levels have been found to correlate with an increased risk of cancer within one year of follow-up, as evidenced by two large population-based studies [61,62]. It is noteworthy that hypercobalaminemia (i.e., elevated B12 levels in the blood) has been detected in both solid tumors and hematological cancers and also linked to a greater risk of prostate cancer [63,64]. Cancer cells often enhance the expression of folate receptors to address their increased demands for cell proliferation [65,66]. Additionally, research from animal studies suggests that administering folate at later stages of colorectal cancer development could promote tumor growth [67]. Similarly, in human studies, a significant chemoprevention trial revealed a surprising 67% increase in advanced colorectal adenomas among patients with a history of adenomas when they received folic acid supplementation (1 mg/day) [68]. These findings underscore the importance of understanding the role of these vitamins in cancer biology, highlighting the need for further research to clarify their effects and guide safer supplementation practices. The mammalian target of rapamycin (mTOR) is the major nutrient sensor of our body; when the availability of dietary energy sources is sufficient, mTOR becomes activated and promotes anabolic metabolism [69,70]. One-carbon metabolism is part of the anabolic metabolism that supports the growth and proliferation of cells [15]. When cellular serine levels decrease, it activates one of the main effectors of the mTOR transcription factor, activating transcription factor 4 (ATF4), which then promotes the expression of serine synthesis pathway (SSP) enzymes to bring the serine level back to normal [71]. Additionally, the mTOR/ATF4 axis was also found to regulate the expression of MTHFD2 and activate the mitochondrial branch of the folate cycle [72]. These studies reinforce the role of mTOR in 1C metabolism and its effects on maintaining cellular homeostasis. Similarly, another study showed that methionine starvation conditions with low SAM levels inhibit mTOR1 signaling in a SAMTOR-dependent fashion [73]. On the other hand, when there are not enough nutrients available to support this anabolic process, AMP-activated kinase (AMPK) is activated to inhibit anabolism and promote the catabolic process to restore ATP levels in the cells [74]. Hence, AMPK acts as an energy sensor; recently, it was shown that AMPK can reduce the flux of 1C units via the expression of MTHFD1/2/1L through the PGC-1α/ERRα axis [75].

## 4. Regulation of 1C Metabolism Under Different Redox Statuses

Oxidants (like free radicals and other reactive species) are continuously produced in different metabolic processes, and our body has different endogenous antioxidants (like GSH, H2S, CAT, etc.) that scavenge and neutralize these oxidants to maintain cellular homeostasis [76]. However, during different disease conditions, this redox homeostasis becomes disrupted, consequently affecting different signaling pathways [77]. The nuclear factor erythroid-derived 2 (NRF2) is a master regulator of redox homeostasis; it has been shown that NRF2 controls the expression of the key enzymes involved in serine and glycine biosynthesis (PHGDH, PSAT1, and SHMT2) [78]. A study using quantitative flux analysis showed that the knockdown of either cytosolic or mitochondrial MTHFD isozyme decreased cellular NADPH/NADP+, reduced/oxidized GSH ratios (GSH/GSSG), and increased cell sensitivity to oxidative stress [79]. This study suggests that other than nucleotide synthesis, another function of 1C metabolism is to develop the reducing power of a cell. Moreover, it was found that besides NAD(P)H production, 1C flux also maintains redox homeostasis via GSH production [80].

GSH is produced from cysteine (via the transsulfuration pathway) via two-step reactions. In the first step, glutamate cysteine ligase (GCL) catalyzes the ATP-dependent condensation of cysteine and glutamate to form the dipeptide gamma-glutamylcysteine (γ-GC) and then, in the second step, γ-glutamylcysteine reacts with glycine to form GSH catalyzed by GSH synthetase (GS). Cysteine is the rate-limiting substrate in this process because the intracellular concentration of glutamate and glycine are higher than the Km values of these reactions [81]. As cystine is produced via the 1C metabolism pathway, any disruption of this pathway may interfere with cellular GSH production.

**Figure 4:**
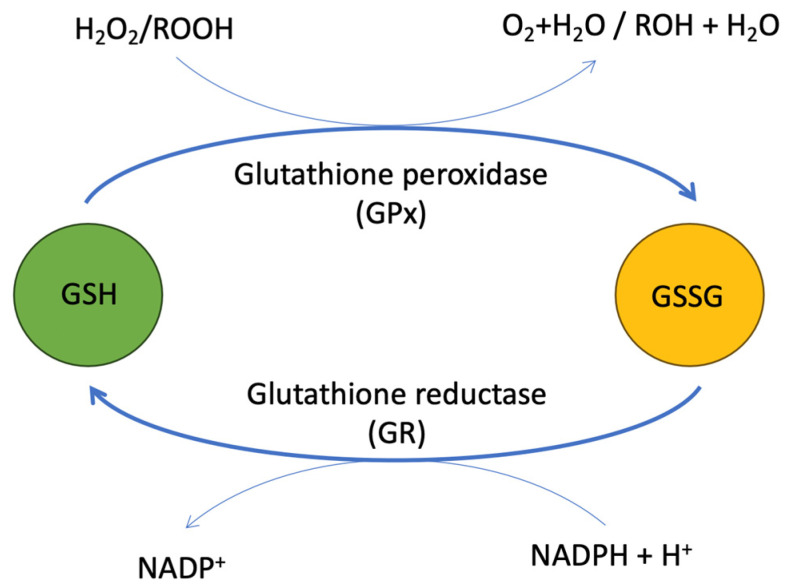
Regulation of redox homeostasis by glutathione (GSH). The enzyme glutathione peroxidase (GPx) uses glutathione (GSH) as a substrate to produce GSSG (oxidized glutathione) by utilizing the thiol (-SH) group of its cysteine residue to interact with reactive oxygen species (ROS) or electrophiles, whereas the enzyme glutathione reductase (GR) efficiently converts GSSG back to GSH with the help of NADPH, thereby preserving the antioxidant capacity of cells.

During the process of electron transfer through the respiratory chain in oxidative phosphorylation, mitochondria play a crucial role in managing reactive oxygen species (ROS). To effectively prevent this ROS (O^2•−^, OH, HOO^•^, H_2_O_2_) from leaking into the cytosol and causing potential harm, mitochondria utilize an antioxidant defense system for ROS scavenging. Different antioxidant enzymes like catalase, peroxiredoxin (Prx), and glutathione peroxidase (GPx) convert these ROS into harmless oxygen and water. After reacting with H_2_O_2_, Prx is reduced back to its functional form through its interaction with thioredoxin (Trx), while oxidized GPx is mainly reduced by glutathione (GSH) [82]. Although GSH is oxidized to glutathione disulfide (GSSG), the enzyme glutathione reductase (GR) ensures the regeneration of GSH from GSSG with the help of nicotinamide adenine dinucleotide phosphate (NADPH) (Figure 4) [83]. The balance of the intracellular redox status is effectively measured by the GSH/GSSG ratio. In healthy conditions, this ratio typically exceeds 100, but it is known to decline to 10 or less during oxidative stress [84,85]. This dynamic regulation highlights how 1C flux also maintains redox homeostasis via GSH production.

## 5. Involvement of 1C Metabolism in Cancer

Cancer cells adapt quickly by changing their metabolism in order to support enhanced cell proliferation and growth [9]. This section discusses how cancer cells become addicted to 1C metabolism for their survival. Due to their high proliferative rate, cancer cells excessively use 1C sources, which are further processed to different compounds, as discussed in the subsequent paragraphs.

### 5.1. Elevated Consumption of 1C Metabolism Precursors in Cancer

#### 5.1.1. High Methionine Utilization in Cancer

One of the primary raw materials of 1C metabolism is methionine, which is required to produce SAM (a methyl group donor), as discussed in Section 2.1 [86]. Cancer cells have high proliferation and growth rates, so they have a high demand for methionine to carry out the methionine cycle [9]. Methionine addition has been known since 1959, when a study by Sugimura et al. showed that the tumor growth rate was considerably reduced when rats were on force-fed diets lacking in methionine amino acid [87]. After that, several studies proposed that compared to non-cancerous cells, many cancer cells stop proliferating when methionine is replaced by its metabolic precursor Hcy [3,88,89,90,91]. This dependence on methionine of cancer cells is known as methionine addiction (a list of methionine-addicted cancer cell lines is summarized in Table 1). A PET study using ^11^C-methionine and ^18^C-fluorodeoxyglucose (FDG) reported that methionine utilization is higher than that of glucose in low-grade glioma [92]. Some studies have also proposed that the high utilization rate of methionine in cancer cells is associated with histone methylations [93].

#### 5.1.2. High Serine and Glycine Utilization in Cancer

In addition to methionine addiction, the elevated consumption of serine and glycine was also noted across the NCI-60 human tumor cell lines [96]. Nonetheless, an isotope tracer analysis found that only serine, not glycine, feeds 1C metabolism in cancer cells in vitro [48]. In normal cell culture conditions, due to the presence of excess methionine and the absence of cobalamin (vitamin B12), homocysteine’s re-methylation was prevented; hence, serine-mediated 1C flux is found only in nucleotide synthesis [97,98].

### 5.2. Elevated Expression of 1C Metabolism Genes in Cancer

**Figure 5:**
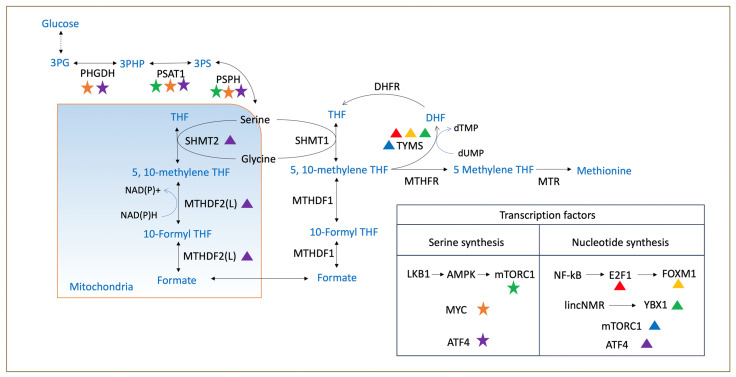
Common transcription factors which are involved in 1C metabolism as well as cancer progression. This visual illustrates the enzymes involved in one-carbon metabolism and their regulating transcription factors, which also play a role in various stages of cancer progression. These transcription factors are organized into two main categories: serine synthesis, represented by different-colored star marks, and nucleotide synthesis, indicated by different-colored triangle marks. All the abbreviations are listed at the end of this article in the abbreviation section.

Consistent with the high demand for raw materials for DNA synthesis to support a high rate of cell proliferation, cancer cells are often found to upregulate the expression of multiple 1C metabolic enzymes [99]. It has been shown that the expression of 1C metabolism pathway genes is controlled by the same transcription factors as those which are involved in cancer progression [53]. It was noted that cancer cells drive cell proliferation, growth, and metastasis by regulating these transcription factors, as shown in Figure 5 [100]. Other than protein expression, different reports also showed genomic amplifications of one of the first enzymes of the serine pathway, PHGDH [101]. Additionally, cancer cells were also found to overexpress the TYMS and DHFR genes involved in the folate cycle [101]. Interestingly, a meta-analysis of tumor gene expression identified that MTHFD2, which is involved in the mitochondrial folate cycle, is overexpressed in multiple cancer types [102].

Moreover, a comparative oncogenomic analysis identified that SHMT2, another mitochondrial gene involved in 1C metabolism, is amplified and is very important for tumor development [103]. These studies highlight the direct involvement of the folate cycle in the DNA replication demands of cancer cells. Surprisingly, another meta-analysis revealed that the expression of enzymes involved in the mitochondrial folate cycle better correlates with sensitivity to methotrexate than the cytosolic folate cycle [104]. Studies also found that the high expression of MTHFD2 is correlated with the migration and invasiveness of breast cancer cells and is associated with a poor prognosis in breast cancer [105,106].

## 6. Involvement of 1C Metabolism in Aging

There is a plethora of evidence which has reported that the dysregulation of 1C metabolism is associated with aging. There are many metabolites which are associated with 1C metabolism and also aligned with age and age-related phenotypes. Research on folate metabolism in young versus aged rats offers valuable insights into the impact of aging on serum folate levels, which decline by approximately 50% [107]. Interestingly, this study found that liver folate content remains stable with age [107]. This suggests that while aging affects the availability of folate in the body, it does not necessarily compromise liver reserves. Mice subjected to a folate-deficient diet from weaning until 8 and 10 months show notable impairments in their circadian rhythms, reminiscent of the changes seen in older adults [108]. Furthermore, such dietary deficiencies manifest as signs of brain aging in these mice, including short-term memory difficulties and alterations in S-adenosylmethionine (SAM) metabolism and acetylcholine levels [108]. On a positive note, supplementing folate within a broader dietary context appears to have beneficial effects on their lifespan [109].

Methionine is an essential amino acid, and recent research has highlighted the potential benefits of reducing its intake for promoting longevity [110,111,112]. Additionally, genetic modifications that induce methionine restriction have yielded similar positive outcomes [113,114]. This insight suggests that methionine restriction (MR) may effectively replicate some of the beneficial metabolic effects associated with caloric restriction (CR). For example, both MR and CR have been shown to lower the production of reactive oxygen species (ROS) in mitochondria, reducing the oxidative damage to mitochondrial DNA (mtDNA) in mice [115].

Elevated plasma levels of homocysteine (Hcy) serve as an important indicator of various age-related diseases, such as cardiovascular issues and stroke [116]. Moreover, both the ubiquitous and neuron-specific overexpression of CBS can lead to increased lifespan in well-fed flies, demonstrating the potential role of CBS in promoting longevity [117]. Functional CBS plays a crucial role in lifespan extension during dietary restriction as well [117,118]. Additionally, overexpressing cbs-1 in the nematode intestine or body wall muscles has been shown to extend the lifespan at both standard (20 °C) and elevated (25 °C) temperatures [119]. These findings underscore the potential benefits of targeting these pathways for promoting health and longevity in various organisms. In the following paragraphs, we discuss role of 1C metabolism in DNA methylation, cellular senescence, and telomere shortening.

### 6.1. Role of 1C Metabolism in Epigenetic Alteration in Relation to Aging

Epigenetics is closely involved in gene transcriptional regulation through modifications super-imposed onto the nucleotide sequence of DNA, such as DNA methylation, through chromatin remodeling systems [120]. In order to regulate gene expression during aging, DNA and histone methylation need methyl donors, such as SAM, from one-carbon metabolism. When this pathway is disrupted, aberrant epigenetic modifications are induced, activating aging-related pathways and contributing to cellular identity loss [21]. Several studies have shown a correlation between the circulating biomarkers of one-carbon metabolism and alterations in nuclear DNA methylation levels in people suffering from age-related diseases. There are increasing numbers of studies demonstrating that even mitochondrial DNA (mtDNA) can be methylated [121]. As mitochondrial activity declines with age, mtDNA methylation may be sensitive to this decline [122]. In one study, Dzitoyeva and colleagues observed that 5-hydroxymethylcytosine levels in mtDNA decreased with aging and a higher expression of genes encoded by mtDNA were seen in the frontal cortex of 4- and 24-month-old mice [122].

### 6.2. Role of 1C Metabolism in Cellular Senescence in Relation to Aging

Cellular senescence has received considerable attention as a factor contributing to aging and disease susceptibility (as described in detail in this section). As evidenced by the identification of senescent cells in multiple tissues with age, senescence plays a key role in the aging process [123], and targeting senescent cells has been found to provide protection in both progeroid models [124] and during normal aging [125].

### 6.3. Role of 1C Metabolism in Telomere Shortening in Relation to Aging

Telomeres are often referred to as an ‘aging clock’ that determines the lifespan of cells. Telomere length can be affected by DNA methylation and, specifically, through DNA’s role in nucleotide biosynthesis, which is essential for telomere maintenance and DNA replication. Insufficient nucleotide availability can cause telomere attrition and the premature aging of cells if this pathway is disrupted. Several review articles have outlined various biological mechanisms through which one-carbon metabolism factors can affect telomere length [126,127,128].

### 6.4. Role of 1C Metabolism in Redox Balance in Relation to Aging

The redox theory of aging presents an intriguing perspective on how longevity may be influenced by the cellular redox state, specifically through the ratios of GSH (glutathione) to GSSG (oxidized glutathione) and of NADP+ to NADPH [129]. This theory highlights the important role of redox buffering systems, which not only protect macromolecules from oxidative damage but also help control the levels of reactive oxygen species (ROS). These ROS function as signaling components, contributing to the aging process [130]. In yeast, research has shown that the depletion of the Gsh1 gene can significantly affect chronological lifespan based on varying dietary glucose levels [131]. Meanwhile, studies in Drosophila melanogaster reveal that enhancing the expression of the catalytic or modulatory subunits of GCL (glutamate-cysteine ligase) can lead to an increase in glutathione levels and promote a longer lifespan [132]. It is important to point out that earlier studies suggested that small amounts of GSH could extend longevity, while more recent investigations have indicated that prolonged supplementation with dietary thiols, such as N-Acetyl Cysteine (NAC) and GSH, may actually accelerate aging [133]. This contrast prompts a closer examination of the mechanisms at play and highlights the complexity of redox biology in aging.

## 7. Involvement of 1C Metabolism in Aging vs. Cancer

It is well-documented that the incidence of cancer is higher in the older age categories compared to younger age categories, which suggests the involvement of a closely interlinked process that may induce more incidences of cancer in the older population [134]. Over the years, extensive research has delved into uncovering the keys to longevity, and these studies suggest the involvement of altered 1C metabolism in this process [135]. It is interesting to notice that folic acid is a crucial micronutrient in one-carbon metabolism, as described in Section 3. It is recommended that pregnant women take folate supplements to support the healthy development of fetal neurons and to help reduce the risk of neural tube defects [136]. The disruption of IC metabolism lies at the heart of age-related diseases like neurodegenerative diseases, cancer, aging, and cardiovascular disease [77,135,137]. During 1C metabolism, via the methylation cycle, Hcy is produced as an intermediate, and excess Hcy levels (i.e., HHcy) have been associated with the aging process via the induction of oxidative stress, lipid peroxidation, DNA damage, etc. [138]. Indeed, studies have shown a prevalence of HHcy in the older population compared to the younger population [139]. Moreover, cysteine, produced during Hcy metabolism via the transsulfuration pathway plays a crucial role in GSH biosynthesis. This GSH acts as a co-substrate for glutathione peroxidase (GPX)-catalyzed reactions, aiding in the removal of hydrogen peroxide (H2O2) and lipid hydroperoxides and generating its oxidized form, glutathione disulfide (GSSG) [15]. Glutathione reductase uses NADPH to facilitate the reduction of GSSG back to GSH. According to the redox theory of aging, the ratios of GSH/GSSG and NADP+/NADPH levels are important factors that may affect longevity by modulating the cellular redox status [140]. The dysregulation of 1C metabolism during aging is associated with the impairment of the oxidative defense mechanism and epigenetic alteration, which further potentiate neoplastic transformation [141]. In the subsequent paragraphs, we discuss how this altered 1C metabolism during aging leads to neoplastic transformation.

### 7.1. Role of 1C Metabolism in Epigenetic Changes in Aging vs. Cancer

The connection between metabolic intermediates and epigenetic regulation has become increasingly important. A phenomenon known as transgenerational inheritance, which suggests that dietary changes can alter the epigenetic landscape, potentially transfers to successive generations [142]. This underscores the importance of metabolites in chromatin dynamics, as well as in all epigenetic modifications, which in turn affect gene expression. Studies have shown that the availability of different nutrients affects the production of Acetyl-CoA, S-adenosylmethionine (SAM), and adenosine triphosphate (ATP), and these metabolites are pivotal for processes like the acetylation, methylation, and phosphorylation of histones [143]. Overall, understanding how metabolites shape epigenetic regulation offers valuable insights into health, with potential implications in aging and cancer.

Histone and DNA methylation are enzymatic reactions that either add or remove methyl groups and regulate the availability of S-adenosylmethionine (SAM) and specific enzymes essential for these processes [144]. SAM acts as a methyl donor and, after the transmethylation reaction, is converted into S-adenosylhomocysteine (SAH), as discussed in Section 2.1. For histones, histone methyltransferases (HMTs) facilitate these reactions [144], whereas for DNA, DNMTs catalyze these reactions [145]. Interestingly, SAH has the potential to inhibit both types of methyletransferases [146,147]. Hence, variations in the SAM/SAH ratio (known as the “Methylation Index”), can influence the activity of various methyltransferases, thus either enhancing or diminishing methylation reactions [148]. Recent advancements have successfully mimicked the chemical structure of SAH, leading to the development of innovative drugs targeting methyltransferases [149]. A prominent example is EZH2, which is significantly overexpressed in several cancer types, including prostate cancer [150]. As a member of the polycomb repressive complex, EZH2 represses transcriptional activity through the methylation of histone H3 at lysine 27 (H3K27 marks) [151]. These developments offer exciting prospects for targeted therapies in cancer treatment, enhancing our understanding of epigenetic regulation. Another enzyme is AHCY, which converts S-adenosylhomocysteine (SAH) to homocysteine and interacts with the CLOCK-BMAL1 complex, playing a key role in circadian regulation [35]. This interaction is crucial for oscillating H3K4me3 marks, suggesting that daily chromatin changes may depend on AHCY activity [152]. Similarly, MAT1A has been shown to join the same chromatin complex, indicating its potential role in one-carbon enzyme regulation, although its specific contributions need further exploration [153]. MAT2A, which converts methionine to S-adenosylmethionine (SAM), interacts with the chromatin complexes critical for gene expression, and particularly that of the MafK-dependent HO-1 gene [153,154]. MAT2A also represses cyclooxygenase 2 (COX2) via its interaction with the H3K9 methyltransferase SETB1, indicating a potential role of 1C metabolism and its association with epigenetic changes [155]. Although these findings hint at important functions for 1C metabolism enzymes in epigenetic alteration, more research is needed to clarify their specific roles and broader impacts within the chromatin landscape.

Changes in DNA and histone methylation are also key indicators of aging. The addition or removal of epigenetic marks relies on enzymatic reactions in the 1C metabolism pathway, with S-adenosylmethionine (SAM) as the universal methyl donor [15]. As studies have shown that the removal of epigenetic marks may speed up the aging process, impairments in this 1C metabolism pathway may decrease epigenetic marks and in turn speeding up aging [156,157]. A study by Kang et al. (2024) compared aged and young mice and found that aged muscle stem cells (MuSCs) had reduced heterochromatin levels, attributed to a depletion of SAM, which prioritized polyamine production over nuclear methylation [158]. Remarkably, supplementing with SAM or inhibiting the polyamine pathway enhanced heterochromatin formation and improved MuSC functionality, offering promising strategies for promoting healthier aging.

### 7.2. Role of Methionine Metabolism in Aging vs. Cancer

Although aging is a natural process, it may induce a cancer risk via the accumulation of various factors, including the loss of telomeres, problems with 1C metabolism, mitochondrial dysfunction, the loss of certain tumor-suppressor genes, and the overactivity of cancer-related genes [159]. As calorie restriction (CR) is the most effective non-genetic intervention at delaying aging, it may reduce cancer risk [160,161]. A preclinical study in rodents indicated that a low-calorie intake is associated with an extended lifespan [162]. Specifically, a 40% CR has been shown to reduce the production of mitochondrial ROS and mitigate oxidative damage to mitochondrial DNA in rodent organs [163]. Past studies have shown that CR decreases ROS production (via reducing NADH levels), thus minimizing oxidative damage and extending the lifespan [164]. Furthermore, various studies have suggested that reducing the intake of 1C metabolites, such as methionine, may be pivotal in extending the lifespan [165]. In rodent studies, it was found that an isocaloric 80% methionine restriction led to increased longevity in rats [166] and mice [167]. Additionally, an 80% methionine restriction was shown to decrease the incidence of age-related degenerative diseases and age-related disease-associated markers, including reductions in serum glucose, insulin, cholesterol, triglycerides, and leptin [43,168,169]. Based on these studies, it is evident that a methionine restriction diet may have a beneficial effect on aging as well as on the development and progression of cancer.

### 7.3. Role of Serine and Glycine Metabolism in Aging vs. Cancer

Like the essential amino acid methionine, some non-essential amino acids involved in 1C metabolism are also connected to aging. In the methionine cycle, during the conversion of SAM to SAH, the methyl group can also be transferred to glycine to form sarcosine (dimethylglycine) via glycine N-methyltransferase (GNMT) [170]. Mice lacking GNMT show a significant increase in free methionine (up to 7-fold) and S-adenosyl-L-methionine (up to 35-fold) [171]. Similarly, studies have reported that glycine supplementation could reduce methionine levels in C. elegans [172] and the overexpression of GNMT could elongate the lifespan of Drosophila [173]. The transfer of a methyl group via GNMT acts as a methionine clearance process, serving as a methionine restriction mimetic, and plays a role in prolonging lifespan [174]. Besides its involvement in the methionine cycle, glycine can act as a building block of GSH production, which may indirectly prolong aging via the reduction of oxidative DNA damage [175]. Interestingly, some studies have also reported that mice lacking GNMT are more prone to hepatocellular carcinoma and steatosis [176].

Similarly to glycine, serine can also affect the aging process via several metabolic regulations [177]. In one process, serine can indirectly influence aging via GSH production through its conversion to glycine [178]. In another mechanism, serine can also produce NADPH, which is found to regulate the senescence process [178]. NADPH levels tend to decrease during aging, and an overexpression of NADPH-synthesizing enzymes is associated with a longer lifespan in certain biological models [179]. These studies suggest that both serine and glycine positively impact the aging process. Aging is a gain-of-function change in cells that may promote their proliferation, migration, and survival, which are also characteristics of cancer cells [180].

### 7.4. Altered Redox Status in Aging vs. Cancer

In the course of aging, different internal factors (e.g., free radicals) and external stressors (e.g., UV radiation, diet, stress, etc.) can heighten genomic instability and, ultimately, lead to oncogenic mutations [181,182]. Reactive oxygen/nitrogen species can react with DNA, causing various types of DNA damage, whereas antioxidant systems such as antioxidant enzymes (e.g., catalase and glutathione peroxidase), vitamins (e.g., vitamins C and E), and other radical scavengers (e.g., glutathione) can prevent these effects [37,183]. Cellular transformation and tumor initiation mainly occur due to oncogenic mutations, often leading to increased growth-promoting signals, disturbed antiproliferative signals, or faulty proapoptotic signaling [184,185]. When cells are damaged due to DNA damage, shortened telomeres, genomic instability, or oncogenic mutations, they can either progress to their apoptotic or senescence stage to suppress tumor development [186]. Apoptosis is a process of programmed cell death that eliminates damaged cells and their subsequent pathology [187], whereas senescence is a process of growth arrest, with cells remaining metabolically active and impacting their neighboring cells in a paracrine fashion [188]. It is still poorly understood which factors determine whether cells will undergo apoptosis, senescence, or both simultaneously.

### 7.5. Antiproliferative Responses During Cellular Damage

Both apoptosis and senescence use cellular defense mechanisms to prevent the progress toward cellular transformation [189]. When these defense mechanisms fail, the DNA damage response is activated, stopping the cell cycle for DNA repair [190]. Essential components of the DNA damage response include ataxia-telangiectasia-mutated (ATM) and ataxia-telangictasia-Rad3-related (ATR) [191]. ATM and ATR activate different mediators to execute the DNA repair, cell cycle arrest, or apoptosis processes [192]. In cases where the DNA repair mechanisms fail, the antiproliferative responses (apoptotic pathway or senescence program) are activated to eliminate cells carrying potentially dangerous mutations that could lead to cancer [193].

**Figure 6:**
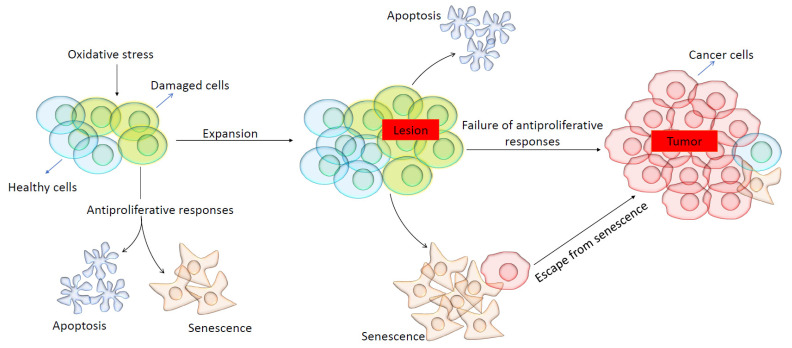
Antiproliferative responses of damaged cells. This cartoon illustrates how damaged cells can become apoptotic, enter senescence, or continue replicating. If these antiproliferative responses are absent or fail, a cancerous lesion may be formed, further proliferating to form malignant cells.

Senescence is a state of irreversible growth arrest but leaves cells metabolically active, serving as a vital defense mechanism against tumor formation [188]. In contrast, apoptosis is a form of programmed cell death that may be used to get rid of damaged cells [194]. If these safeguards fail, the cells may continue replicating and potentially develop into a lesion [195]. At this stage, apoptotic or senescence programs can be reactivated, as shown in Figure 6 [195]. However, if these protective measures fail, the lesion can grow and accumulate genetic and epigenetic abnormalities, ultimately leading to the development of a malignant tumor [196]. It is important to note that despite the induction of cellular senescence or apoptosis, cells can sometimes bypass these processes and transform into malignant cells [197].

Cellular senescence is widely recognized as a key factor in the aging process, and it can be activated by factors such as oxidative stress, telomere shortening, and oncogene expression [198]. It is important to notice that the two most crucial cellular senescence genes, TP53 and P16INK4a, are also tumor-suppressor genes [199]. The TP53 gene produces the P53 protein, which activates the CDK inhibitor P21, ultimately inhibiting CDK4/6 activity [200,201]. Similarly, the P16INK4a gene, part of the CDKN2a/INK4a/ARF locus, generates two protein products, one of which regulates P53 stability, while the other, the P16INK4a protein, inhibits CDK4/6 [202]. Both P53 and P16INK4a prevent the activation of CDK4/6, leading to the inhibition of retinoblastoma protein (pRB) phosphorylation and subsequent cell cycle arrest in the G1 phase [203].

Cellular senescence is a critical process for normal development and maintaining tissue balance [159]. However, it can have adverse effects as cells age [204]. In a mouse experiment, researchers found that mice with an activated TP53 gene exhibited both increased tumor resistance and early aging traits, indicating the complex role of TP53 in cellular function [205]. Moreover, the study reinforced the importance of P53 in eliminating telomere-damaged cells and its impact on aging [206]. Similarly, knocking out P16INK4a in mice led to increased cancer development and reduced survival, highlighting the protective role of this gene [207]. Additionally, elephants’ enhanced tumor resistance due to their extra copies of the TP53 gene further emphasizes the cancer-protective role of TP53 [208].

## 8. The Decision of a Cell’s Fate as Senescence or Apoptosis

**Figure 7:**
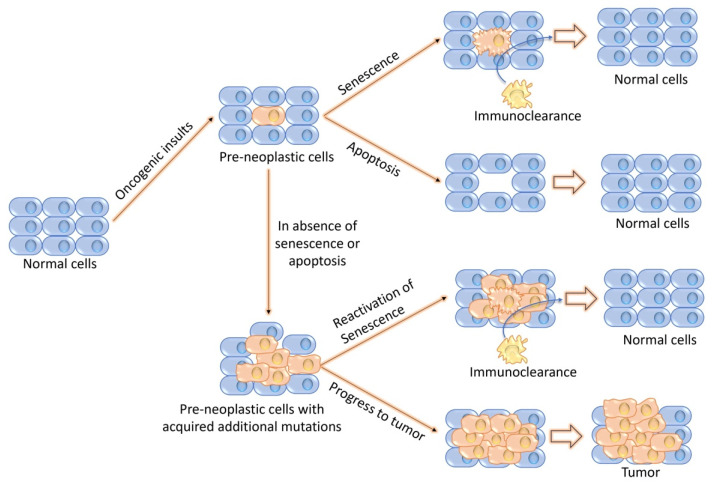
Schematic representation of the fate of cells undergoing senescence and apoptosis upon oncogenic insults. In response to various stressors, normal cells with pre-neoplastic lesions may undergo senescence or apoptosis with the final goal of removing the pre-neoplastic cells. However, in the absence of these antiproliferative responses, pre-neoplastic cells continue to grow and acquire additional oncogenic mutations. At this step, senescence can be reactivated, or it can progress toward malignant transformation.

During the course of the aging process, cells acquire pre-neoplastic lesions via free radicals as the products of metabolic reactions or as the byproducts of various cellular processes [209]. Cells that have acquired a pre-neoplastic lesion may undergo senescence or apoptosis, as shown in Figure 7. Although the final outcomes of these two processes are the same, if senescence begins, it ensures that the lesion is efficiently removed via immunosurveillance, whereas, if apoptosis begins, it destroys the damaged cells without initiating an immune response [195]. In contrast, if the pre-neoplastic lesion dose does not initiate either senescence or apoptosis, these cells continue to grow and form a malignant phenotype [195]. Theoretically, the senescence process entails the complications of fueling inflammation and disrupting the tissue’s structure and function; why do biological systems not rely on apoptosis alone? Many senescence-inducing stressors are linked to oncogenic factors, indicating that senescence and apoptosis may have evolved in tandem to inhibit tumor development [210]. There may be an evolutionary pressure to protect cells that are exposed to mutation-causing influences. Senescence induction may be a favorable process for responding to a mutagenic stress that stops cell growth (and the resulting cancer threat) and remaining alive. It has been observed that normal skin melanocytes produce significant levels of the anti-apoptotic protein BCL2, which plays a crucial role in promoting melanocyte survival [211,212]. Additionally, senescent fibroblasts exhibit a resistance to apoptosis by expressing high levels of BCL2 [213]. Notably, mouse lymphomas expressing BCL2 undergo drug-induced senescence, highlighting the potential significance of BCL2 in this process [214].

As no biological processes have been identified that reverse senescence growth arrest, this process is considered irreversible [215]. Although cellular senescence during embryogenesis is a transient growth arrest and programmed process that plays a role in tissue remodeling, in adult tissue, senescence is considered a permanent growth arrest [204]. These in vitro findings sparked speculation about potential in vivo correlates that could protect against cancer. However, it is ironic that senescent cells can actually fuel malignant phenotypes and tumor growth [216]. It is worth noting that senescent cells, particularly those that become senescent in response to DNA-damaging agents (like radiation and chemotherapeutic agents), release substances that have the potential to protect neighboring tumor cells from being targeted and eliminated by the same chemotherapeutic agents [217].

Recent findings suggest that cellular senescence has beneficial effects on tumors. In addition to arrested growth, senescent cells were found to secret numerous pro-inflammatory cytokines, chemokines, growth factors, and proteases (known as senescence-associated secretory phenotype or SASP) [218]. It is interesting to identify that certain SASP components can apparently act in an autocrine fashion to block tumor growth [219]. Specific SASP components like IL-6, IL-8, and IGFBP7 in human cells strengthen the growth arrest of senescence caused by oncogenic forms of RAS and BRAF [216]. Similarly, GROα, a SASP component induced by oncogenic RAS, encourages the senescence of normal human ovarian fibroblasts [216]. For instance, only a small number of moles, which are benign tumors of cutaneous melanocytes and contain oncogenic mutations, grow larger than 1 cm, and fewer than 1 in 1000 of these progress to melanoma [220]. Additional work will be required to reveal the contribution of other factors to the cessation of neoplastic progression in such lesions.

## 9. Conclusions

It is a long-standing question in oncology as to why cancer develops later in life. One potential explanation is that metabolic impairment, epigenetic changes, and oncogenic mutations during the aging process drive the formation of tumors [221]. One study indicates that both copies of the autosomal tumor suppressor gene must be inactivated to form a tumor, and metabolite changes during aging play a significant role in this process [222]. Another report also suggests that even a transient epigenetic event can lead to tumorigenesis without additional stimuli, potentially inducing tumors without driver mutations [223]. While current data in support of the above hypothesis are limited, further studies may establish such a connection. Cellular functions are known to be tightly regulated by metabolic processes [224]. Embracing a systems biology approach, rather than focusing solely on individual genes or metabolic pathways independently, holds immense potential [225]. This approach offers a promising avenue for future research to uncover the critical interplay between 1C metabolite levels, metabolic enzymes, subcellular localization, and changes in the chromatin landscape.

## Figures and Tables

**Table 1:** List of methionine-addicted cancer cell lines.

Types	Name of the Cell Lines	References
Breast Cancer	MDA-MB468	[91,94]
Breast Cancer	MDA-MB361	[91]
Breast Cancer	MCF7	[3,90,94]
Breast Cancer	HCC1806, HCC1143, SKBR3, BT-549, ZR-75-1, SUM-159, T47D	[94]
Breast Cancer	W-256	[88,89]
Colon cancer	SK-CO-1	[3]
Prostate cancer	PC-3, DU145	[3,90,95]
Prostate cancer	LNCaP	[95]
Lung cancer	A2182, SK-LU-1	[3,90]
Lung cancer	A549	[3]
Bladder cancer	J82, T24	[3,90]
Melanoma	A375	[3]
Cervical cancer	HeLa	[3]
Kidney cancer	A498	[3,90]
Glioblastoma	A172	[3,90]
Neuroblastoma	SK-N-SH	[3,90]
Rhabdomyosarcoma	A673, A204	[3,90]
Osteosarcoma	HOS	[3,90]
Fibrosarcoma	HT1080, 8387	[3,90]
Monocytic leukemia	J111	[89]
Lymphatic leukemia (mouse)	L1210	[89]
Transformed fibroblast	SV80	[88]
SV40-transformed human cells	W18VA2	[88]

## Abbreviations

1C	one-carbon
OXPHOS	oxidative phosphorylation
ROS	reactive oxygen species
THF	tetrahydrofolate
NADH	nicotinamide adenine dinucleotide
NADPH	nicotinamide adenine dinucleotide phosphate
SHMT	serine hydroxymethyltransferase
PSPH	phosphoserine phosphatase
PSAT1	phosphoserine aminotransferase 1
PHGDH	phosphoglycerate dehydrogenase
DHF	dihydrofolic acid
PET	positron emission tomography
Hcy	homocysteine
HHcy	hyperhomocysteinemia
GSSG	glutathione disulfide
ATM	ataxia-telangiectasia-mutated
ATR	ataxia-telangictasia-Rad3-related
CDK	cyclin-dependent kinases
Bcl-2	B-cell leukemia/lymphoma 2
IL	interleukin
IGFBP7	insulin-like growth factor binding protein 7
SASP	senescence-associated secretory phenotype
TYMS	thymidylate synthase
PPP	pentose phosphate pathway
dUMP	deoxyuridine monophosphate
dTMP	deoxythymidine monophosphate
GCS	glycine cleavage system
ATF4	activating transcription factor 4
AMPK	AMP-activated kinase
3-PG	3-phosphoglycerate
CSE	cystathionine γ-lyase
GSH	glutathione
PSAT	phosphoserine aminotransferase
MTHF	5-methyltetrahydrofolate
B_12_	vitamin B_12_
B_6_	vitamin B_6_
BHMT	betaine–homocysteine S-methyltransferase
CBS	cystathionine β-synthase
dcSAM	decarboxylated SAM
DMG	dimethylglycine
E1	enolase-phosphatase 1
G/AT	glutamine or asparagine transaminase
GNMT/DNMT1	glycine N-methyltransferase or DNA methyltransferase 1
MTA	methylthioadenosine
ERRα	estrogen-related receptor alpha
PGC-1α	peroxisome proliferator-activated receptor gamma coactivator 1-alpha
MAT	methionine adenosine transferase
MTAP	methylthioadenosine phosphorylase
MTOB	methylthiooxobutyrate
MTR	methionine synthase
MTRR	methionine synthase reductase
MTRD	methylthioribulose dehydratase
MTNA	methylthioribose isomerase
ODC	ornithine decarboxylase
SAH	S-adenosylhomocysteine
SAHH	SAH hydroxylase
SAM	S-adenosylmethionine
SAMDC	SAM decarboxylase
SMS	spermine synthase
SRM	spermidine synthase
ATF4	activating transcription factor 4
AMPK	AMP-activated protein kinase
E2F1	2F transcription factor 1
FOXM1	forkhead box M1
LKB1	liver kinase B1
lincNMR	long intergenic noncoding RNA-nucleotide metabolism regulator
mTORC1	mechanistic target of rapamycin complex 1
NF-κB	nuclear factor–kappa B
YBX1	Y-box binding protein 1
